# Ethylene enhances resistance to cucumber green mottle mosaic virus via the *ClWRKY70*-*ClACO5* module in watermelon plants

**DOI:** 10.3389/fpls.2023.1332037

**Published:** 2024-01-11

**Authors:** Mei Liu, Baoshan Kang, Huijie Wu, Bin Peng, Liming Liu, Ni Hong, Qinsheng Gu

**Affiliations:** ^1^ Zhengzhou Fruit Research Institute, Chinese Academy of Agricultural Sciences,Henan Key Laboratory of Fruit and Cucurbit Biology, Zhengzhou Henan, China; ^2^ Key Lab of Plant Pathology of Hubei Province, College of Plant Science and Technology, Huazhong Agricultural University, Wuhan, China; ^3^ Zhongyuan Research Center, Chinese Academy of Agricultural Sciences, Xinxiang, Henan, China; ^4^ Institute of Plant Protection, Xinjiang Academy of Agricultural Sciences, Xinjiang, China

**Keywords:** ethylene, *ClACO5*, cucumber green mottle mosaic virus, *ClWRKY70* regulation, watermelon

## Abstract

**Introduction:**

Ethylene (ET) is involved in plant responses to viral infection. However, its molecular mechanisms and regulatory network remain largely unknown.

**Methods and results:**

In the present study, we report that cucumber green mottle mosaic virus (CGMMV) in watermelon (*Citrullus lanatus*) triggers ET production by inducing the expression of *ClACO5*, a key gene of the ET biosynthesis pathway through transcriptome data analysis and gene function validation. The knock-down of *ClACO5* expression through virus-induced gene silencing in watermelon and overexpressing *ClACO5* in transgenic *Nicotiana benthamiana* indicated that ClACO5 positively regulates CGMMV resistance and ET biosynthesis. The salicylic acid-responsive transcription factor gene *ClWRKY70* shares a similar expression pattern with *ClACO5*. We demonstrate that *ClWRKY70* directly binds to the W-box *cis*-element in the *ClACO5* promoter and enhances its transcription. In addition, *ClWRKY70* enhances plant responses to CGMMV infection by regulating *ClACO5* expression in watermelon.

**Discussion:**

Our results demonstrate that the *ClWRKY70*-*ClACO5* module positively regulates resistance to CGMMV infection in watermelon, shedding new light on the molecular basis of ET accumulation in watermelon in response to CGMMV infection.

## Introduction

Plants constantly face challenges from various biotic stressors, including viruses, that affect plant growth and development and cause crop losses, posing serious threats to food security (Calil and Fontes, 2017). Upon infections, the physiological and biochemical changes in the host plant are triggered in virus-host interactions. These alterations might lead to plant exhibition of disease symptoms such as mottling, a mosaic, wrinkling of leaves, plant dwarfing, and fruit decay (Mishra et al., 2020). To counter defense against viruses, plants have evolved complex, delicate defense systems to restrict viral infection, including gene silencing, regulation of metabolism, and phytohormone-mediated defense (Incarbone and Dunoyer, 2013). Among them, the accumulation of plant hormones such as salicylic acid (SA), jasmonate (JA), ethylene (ET), and abscisic acid can increase plant resistance to viral infection (Zhu et al., 2014a; Casteel et al., 2015; Alazem and Lin, 2017; Zhang et al., 2017).

In plant development, ET participates in a series of physiological processes, such as seed germination and plant senescence, as well as defense against necrotrophic pathogens (Pieterse et al., 2012; Zhao et al., 2017). While in terms of virus defense, ET could be both a positive and a negative factor. For example, the rice dwarf virus (RDV) increases ET production by interacting with OsSAMS1, an essential component of the ET biosynthesis, resulting in an increase in the enzyme’s activity, thereby increasing susceptibility to RDV (Zhao et al., 2017). Among ET pathway mutants in *Arabidopsis*, *ethylene insensitive 2* (*ein2*) and *ethylene response 1* (*etr1*) show improved resistance to cauliflower mosaic virus (CaMV) infection, and *1-aminocyclopropane-1-carboxylate synthase* (*acs6*) and *ethylene-responsive transcription factor 104* (*erf106*) show enhanced resistance to tobacco mosaic virus (TMV-cg) infection in crucifers (Love et al., 2007; Chen et al., 2013). By contrast, manipulation of JA and ET signaling in tobacco carrying the resistance gene *N* conveyed systemic resistance to chilli veinal mottle virus (Zhu et al., 2014a).

ET is synthesized via a two-step reaction: first, ACC synthase (ACS) converts *S*-adenosylmethionine (SAM) into 1-aminocyclopropane-1-carboxylic acid (ACC), which is then oxidized by ACC oxidase (ACO) to produce ET (Yang and Hoffman, 2003). ACO is a member of the DOXC subclass of the plant 2-oxoglutarate-dependent dioxygenase superfamily (Kawai et al., 2014). *ACO* genes belong to multigene families in most plants (Booker and DeLong, 2015; Houben and Van de Poel, 2019). Only a few ACOs have been proven to play a role in ET biosynthesis and plant responses to pathogen infection (Kim et al., 1998; Yim et al., 2014; Zeilmaker et al., 2015; Houben and Van de Poel, 2019). *ACO* genes are under tight regulation and show tissue-specific expression and localization patterns (Blume and Grierson, 1997; Van de Poel et al., 2012; Pattyn et al., 2021). However, only a few transcription factors (TFs) that modulate *ACO* expression have thus far been identified (Houben and Van de Poel, 2019).

The host gene expressions are altered at transcription to posttranslational level in plant immunity responses at any stage of development. Environmental and/or endogenous cues might activate TFs that bind to the regulatory regions of their target genes, thereby enhancing or inhibiting their transcription (Fan et al., 2014; Chen et al., 2017; Chen and Penfield, 2018; Chen et al., 2018; Zhou et al., 2018; Liu et al., 2021). One of the largest plant TF families, WRKY TFs, is crucial for plant immunity (Kalde et al., 2003; Zhou et al., 2018). WRKY TFs include at least one conserved WRKY domain that binds to W-box motifs in their target genes’ promoters (Hu et al., 2013). For example, *WRKY72* increases the susceptibility of rice (*Oryza sativa*) to bacterial blight by binding to WRKY-binding motifs in the promoter of *AOS1* and inhibiting its expression, reducing JA levels (Hou et al., 2019). *WRKY8* directly binds to the W-box *cis*-element in their promoters, regulating the expression of *ACS6, ABI4, and ERF104*, thus participating in the defense response against TMV-cg (Chen et al., 2013). *WRKY70* functions in SA-dependent defense and as a repressor of JA-regulated genes and enhances host defense responses against pathogen infection by activating the expression of many defense-related genes, including *PR1*, *PR2*, *PR5*, and *SARD1 (* Li et al., 2004). Notably, a link between *WRKY70*, *ACO*, and ET accumulation has not been identified, although they are individually associated with plant responses to pathogens.

As an important horticultural crop globally, watermelon (*Citrullus lanatus*) is vulnerable to a variety of viral diseases. For example, the cucumber green mottle mosaic virus (CGMMV), a species in the genus *Tobamovirus*, seriously affects watermelon yields and quality (Dombrovsky et al., 2017). No commercial CGMMV-resistant cultivars are currently available. Moreover, little is known about CGMMV resistance genes in watermelons (Cai et al., 2023). Identifying CGMMV-responsive genes might allow for the exploration of the molecular mechanisms underlying CGMMV resistance in watermelon plants, which benefits the molecular breeding of watermelons.

We previously performed transcriptome sequencing to explore the global transcriptome of watermelon leaves systemically infected with CGMMV and found a few differentially expressed genes (DEGs) related to various metabolic pathways (Liu et al., 2023a). In this study, we identified *ClACO5*, an *ACO* gene of watermelon, and evaluated its role in ET synthesis and CGMMV resistance. Watermelon WRKY70 (ClWRKY70) promotes its transcription by directly binding to the *ClACO5* promoter. In addition, *ClWRKY70* expression is regulated by SA, and ClWRKY70 positively regulates plant defense against CGMMV. Hence, we identified a *ClWRKY70*-*ClACO5* regulatory module that governs CGMMV-induced *ClACO5* upregulation and ET biosynthesis. This finding reveals the molecular basis of ET accumulation in the response of watermelon to CGMMV infection.

## Results

### CGMMV induces ET accumulation, and ET treatment enhances CGMMV resistance in watermelon

**Figure 1:**
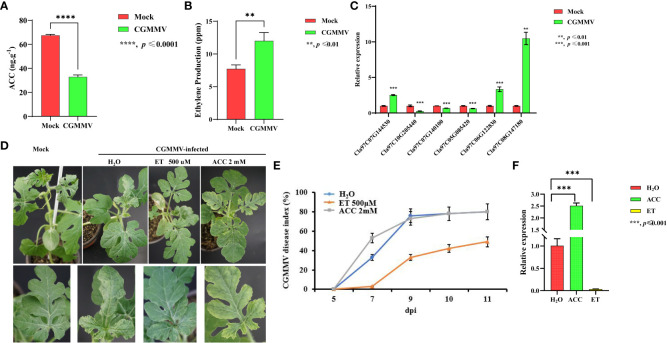
CGMMV induces ET accumulation, and exogenous ET enhances the resistance of watermelon plants to CGMMV. **(A, B)** ACC **(A)** and ET **(B)** levels in mock- and CGMMV-treated watermelon leaves. **(C)** The transcript levels of ET pathway genes in mock-inoculated or CGMMV-infected watermelon leaves at 10 dpi by RT-qPCR. **(D)** Phenotypes of 7-day-old seedlings treated with water, ET (500 μM), or ACC (2 mM) before CGMMV treatment. **(E)** CGMMV disease indices of mock-inoculated or CGMMV-infected watermelon plants at different time points. **(F)** RT-qPCR of CGMMV CP accumulation in water-, ET (500 μM)-, or ACC (2 mM)-treated watermelon plants. Plants were collected at 10 dpi. Values are means ± SD (n = 3). **p ≤ 0.01, ***p ≤ 0.001 (Student’s *t*-test).

We previously showed that biosynthesis of the ET precursor ACC is inhibited by CGMMV infection in a susceptible watermelon variety, enhancing its susceptibility to viral infection (Liu et al., 2023a). To explore the role of the ET pathway in plant defense against CGMMV infection, we measured the concentrations of ACC and ET in mock- and CGMMV-infected plants of a susceptible watermelon variety (Zhengkang No. 2) at 10 days post-inoculation (dpi) (**Figures 1A, B**). At this stage, we observed typical symptoms, including mottle and mosaic patterns on leaves, in CGMMV-infected watermelons. In addition, CGMMV RNAs accumulated to high levels in these plants (Liu et al., 2023a). ACC levels in CGMMV-infected plants were about half of those in mock-inoculated plants (**Figure 1A**), whereas ET levels were approximately twice as high in CGMMV-infected as in mock-inoculated plants (**Figure 1B**).

We wondered whether ACC and ET play opposite roles in antiviral defense in watermelons. To address this question, we sprayed watermelon leaves with ACC (2 mM), ET (500 µM), or H_2_O (as a control) 72 h before inoculation of CGMMV. At 10 dpi, ACC-treated plants were more susceptible to CGMMV infection, displaying more severe disease symptoms, higher disease indices, and higher levels of viral accumulation than H_2_O-treated plants (**Figures 1D-F**). However, ET-treated plants showed that resistance to CGMMV was enhanced, with the exhibition of milder disease symptoms, lower disease indices, and lower levels of virus accumulation, compared to H_2_O-treated plants (**Figures 1D-F**). These results undoubtedly suggest that ACC increases the susceptibility of watermelon to CGMMV infection, whereas ET enhances its resistance to this virus.

### Identification and analysis of *ClACO5*

To identify the genes of watermelon responsible for ET accumulation upon CGMMV infection, we analyzed the gene expression profiles of mock- and CGMMV-infected watermelon seedlings by RNA sequencing (RNA-Seq) (Liu et al., 2023a). The expression levels of >1,200 genes significantly altered upon CGMMV infection (Liu et al., 2023a). Gene Ontology (GO) analysis showed that these DEGs were enriched for biological processes in the ET response category (**Supplementary Figure S1A**). In addition, the expression levels of approximately two-thirds of the 30 ET-related genes were altered in CGMMV-infected watermelons, including genes encoding ET synthases (*ACO* genes), ET receptors (*ETR* genes), and ET-responsive TFs (*ERF* genes) (**Supplementary Figure S1B**). Specifically, the expression of most *ERF*, *ACO*, and *ETR* genes was inhibited (**Figure 1C** and **Supplementary Figure S1B**), whereas the expression of only one *ACO* (Cla97C07G144530) and two *ERF* genes (Cla97C06G122830 and Cla97C08G147180) was induced by CGMMV infection. Quantitative reverse-transcription PCR (RT-qPCR) further confirmed the results of RNA-Seq data (**Figure 1C**). All those results suggest that these ET-related genes might contribute to antiviral defense responses in watermelons.

Three DEGs annotated as *ACO* genes displayed marked changes in expression (**Figure 1C**), with only one (Cla97C07G144530) being upregulated upon CGMMV infection. We, therefore, explored whether this gene might be related to the decrease of ACC content and increase of ET content upon CGMMV infection. Sequence analysis showed that the cDNA amplified from the contig included a 1,038-bp open reading frame encoding a protein of 345 amino acids with a predicted molecular mass of 39.7 kDa and an isoelectric point of 6.07. BLAST analysis and sequence alignment of Cla97C07G144530 with its homologs revealed that its predicted amino acid sequence shares 87.54%, and 65.31% amino acid identity with CsACO5 (1-aminocyclopropane-1-carboxylate oxidase 5, XP_004137205) in cucumber (*Cucumis sativus*) and AtDMR6 (AT5G24530.1) in *Arabidopsis thaliana* (**Supplementary Figure S2**), respectively. We named the Cla97C07G144530 gene as *ClACO5*.

### ClACO5 localizes to the cytoplasm and nucleus

**Figure 2:**
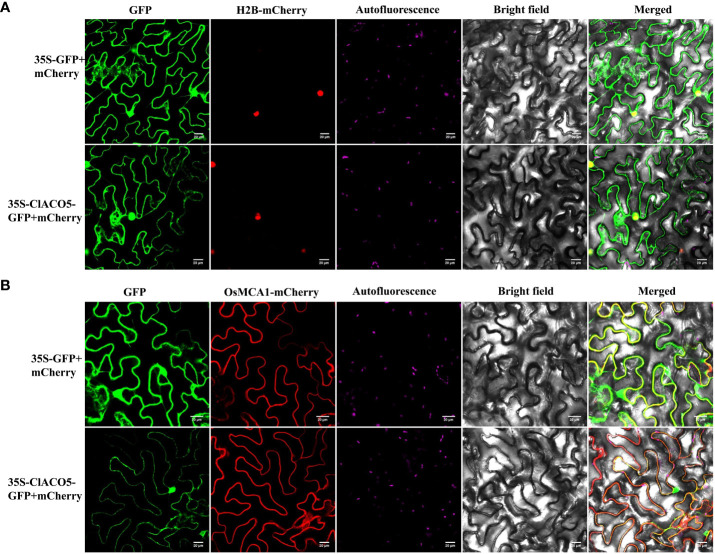
Subcellular localization of ClACO5 protein. Control vector (35S-GFP) or ClACO5-GFP fusion protein (35S-ClACO5-GFP) were co-transformed with a nuclear marker protein H2B **(A)** and membrane marker protein OsMCA1 **(B)** fused to mCherry (red fluorescent protein, 35S-H2B-mCherry or 35S- OsMCA1-mCherry) in tobacco (*Nicotiana benthamiana*) leaves. Bars, 20 μm.

Bioinformatics prediction revealed that ClACO5 might be localized to the cytoplasm (**Supplementary Figure S3**). To confirm this prediction, 35S-ClACO5-GFP or 35S-GFP was co-transformed with a nuclear marker protein H2B (35S-H2B-mCherry) or a membrane marker protein OsMCA1 (35S-OsMCA1-mCherry) into *Nicotiana benthamiana* leaves. The GFP signals in leaf cells expressing 35S-ClACO5-GFP or 35S-GFP completely overlapped with the signals of mCherry (**Figure 2**), suggesting that ClACO5 was localized in the cytoplasm and nucleus of *N. benthamiana*.

### *ClACO5* positively contributes to CGMMV resistance

**Figure 3:**
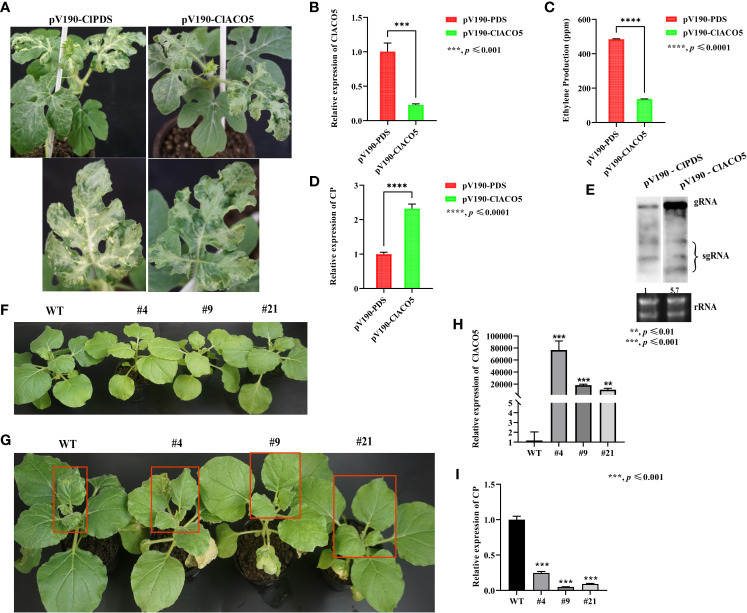
Knockdown and overexpression of ClACO5 enhance CGMMV susceptibility and resistance in watermelon plants and transgenic *N. benthamiana*. **(A)** Phenotype of pV190-ClPDS and pV190-ClACO5 in watermelons. **(B)** The transcript levels of *ClACO5* in pV190-ClPDS- and pV190-ClACO5-treated watermelon leaves at 20 dpi by RT-qPCR. **(C)** Ethylene production of pV190-ClPDS- and pV190-ClACO5-treated watermelon leaves at 20 dpi. **(D, E)** CGMMV RNA accumulations in the pV190-ClPDS- and pV190-ClACO5-treated watermelon leaves at 20 dpi by RT-qPCR **(D)** and Northern blotting **(E)**. **(F)** Phenotype of WT and transgenic *N. benthamiana* plants under normal growth conditions. **(G)** Phenotype of WT and transgenic *N. benthamiana* plants under CGMMV infection conditions. **(H)** RT-qPCR analysis of the transcript levels of *ClACO5* in WT and transgenic tobacco plants. **(I)** RT-qPCR analysis of CGMMV CP accumulations in transgenic and WT tobacco plants. Watermelon and *N. benthamiana* plants were all collected at 20 dpi and 10 dpi, respectively. Values are means ± SD (n = 3). **p ≤ 0.01, ***p ≤ 0.001, and ****p ≤ 0.0001 (Student’s *t*-test). Image of northern blotting was obtained by splicing, as these two samples and other samples were hybridized on the same membrane simultaneously.

We generated ClACO5-knockdown watermelon plants (pV190-ClACO5) via agroinfiltration by our previously constructed pV190-based virus-induced gene silencing (VIGS) system (**Figure 3A**). *ClACO5* expression levels of silenced plants were only 23% of that in control plants at 20 dpi (**Figure 3B**), as well as demonstrated less ET accumulation than controls (**Figure 3C**). To examine the impact of *ClACO5* downregulation on CGMMV accumulation, we used systemically infected leaves from control (phytoene desaturase [*PDS*]-silenced) and *ClACO5*-silenced watermelon plants to measure CGMMV RNA levels. The accumulation of CGMMV gRNA in ClACO5-knockdown plants increased by 230% and 570% compared to that in *PDS*-knockdown plants, as revealed by RT-qPCR and RNA gel blot analysis, respectively (**Figures 3D, E**). These findings manifest that ClACO5 increases ET levels and enhances defense against CGMMV in watermelon plants.

To further confirm that ClACO5 functions in plant defense against CGMMV infection, we exogenously overexpressed *ClACO5* in *N. benthamiana* (a CGMMV-susceptible species) and selected three transgenic lines to assess CGMMV resistance. Under normal growth conditions, the wild-type (WT) and transgenic tobacco plants did not exhibit phenotypic differences (**Figure 3F**), and *ClACO5* transcript abundance was significantly higher in the transgenic plants (**Figure 3H**). However, upon CGMMV infection, these plants showed milder CGMMV disease symptoms, including milder mottle and mosaic symptoms in systemically infected leaves, than the WT plants (**Figure 3G**). In addition, transgenic *N. benthamiana* plants accumulated significantly less CGMMV than the WT plants (**Figure 3I**). Collectively, these results show that knocking down *ClACO5* significantly enhanced CGMMV susceptibility, whereas overexpressing this gene enhanced resistance to this virus, indicating that ClACO5 plays a positive role in ET biosynthesis.

### *ClWRKY70*, a transcriptional activator, is induced by CGMMV infection

**Figure 4:**
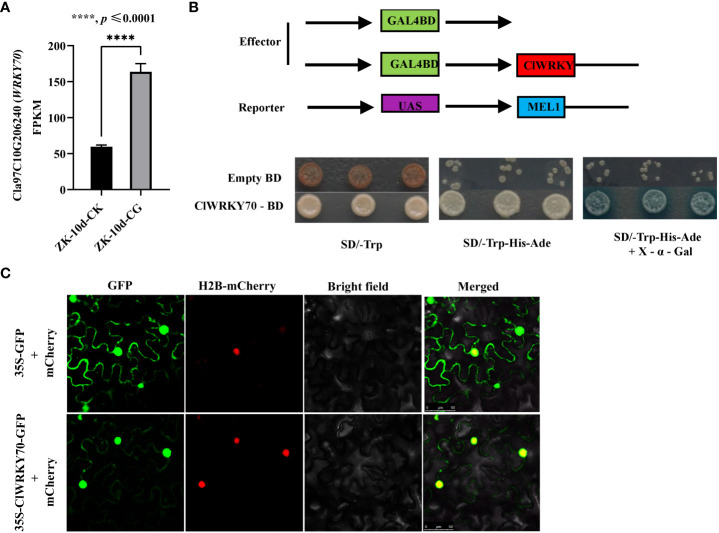
*ClWRKY70* expression under CGMMV infection and transcriptional activity assays. **(A)** *ClWRKY70* expression level was assayed after CGMMV infection. **(B)** Transactivation assay of *ClWRKY70* in yeast. **(C)** Subcellular localization of ClWRKY70 in tobacco. 35S-ClWRKY6-GFP or 35S-GFP was co-transformed with 35S-H2B-mCherry (carried nuclear marker protein H2B) into tobacco leaves. The images of epidermal cells were taken under green (for GFP) and red (for mCherry) fluorescence and bright fields by confocal microscopy. Bars, 50 μm. ZK-10d-CK, ZK-10d-CG, leaves of ‘ZK’ plants inoculated with CGMMV or 0.01 M phosphate butter, were harvested at 10 dpi.

To identify TFs that regulate *ClACO5* expression, we conducted a manual search of the NewPLACE database for the *cis*-elements in the *ClACO5* promoter sequence (~2 kb) involved in responses to plant pathogens. Several W-boxes and MYB motifs in the *ClACO5* promoter were identified, which are recognized by WRKY and MYB transcription factors, respectively. An abscisic-acid-response element (ABRE), an SA-responsive element, and a methyljasmonate (MeJA)-responsive element were also found in the *ClACO5* promoter (**Supplementary Table S1** and **Supplementary Figure S4**). Analysis of the RNA-Seq data found that CGMMV also induced *ClWRKY70* (Cla97C10G206240) expression and that this gene shared similar expression patterns with *ClACO5* (**Figure 4A**). Next, we examined the subcellular localization of ClWRKY70 by transiently co-expressed 35S-ClWRKY6-GFP or 35S-GFP with the nuclear marker gene *H2B* fused to mCherry in *N. benthamiana* leaves. The GFP signal from the 35S-GFP vector was ubiquitously distributed throughout the cell. By contrast, the GFP signal from the 35S-ClWRKY70-GFP vector was exclusively localized to the nucleus and co-localized with mCherry (**Figure 4C**). To examine the transcription-activating activity of ClWRKY70, a transactivation reporter assay was performed. Yeast strain AH109 containing pGBKT7-ClWRKY70 grew well on SD/–Trp or SD/–Trp/–Ade/–His medium, and displayed GAL4 activity on SD/–Trp/–Ade/–His medium supplemented with X-α-Gal, suggesting that ClWRKY70 is a transcriptional activator (**Figure 4B**).

### ClWRKY70 binds to the *ClACO5* promoter

**Figure 5:**
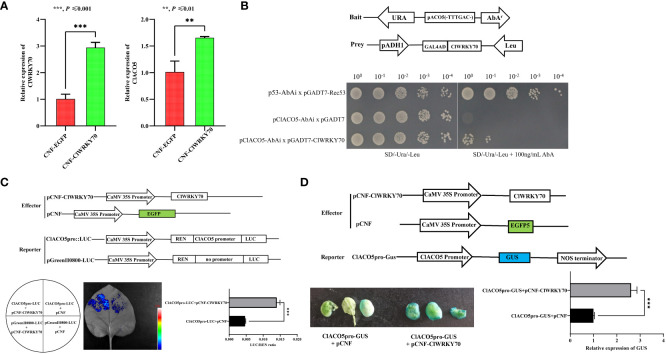
*ClWRKY70* activates the transcription of *ClACO5*. **(A)** The transcript level of *ClWRKY70* and *ClACO5* after transient overexpression of *ClWRKY70* in watermelon leaves. **(B)** Yeast one-hybrid (Y1H) analysis using pGADT7-ClWRKY70 as prey, pClACO5-AbAi as bait, and pGADT7-Rec53 and p53-AbAi as positive controls. **(C)** Luciferase activity analysis showed that higher LUC/REN ratio in the pCNF-ClWRKY70 effect vector than with the pCNF effect vector. The pCNF-ClWRKY70/pCNF effector and ClACO5pro-LUC/pGreenII800-LUC reporter were co-infiltrated into tobacco leaves. **(D)** GUS activity analysis using ClACO5pro-GUS as reporters and pCNF-ClWRKY70 as an effector. GUS staining images and GUS gene expression levels were visualized and measured.

CGMMV infection increased the abundance of *ClWRKY70* and *ClACO5* transcripts; these genes play vital roles in plant responses to pathogen infection. We also identified several WRKY-binding motifs in the *ClACO5* promoter (**Supplementary Table S1**). These observations prompted us to examine whether ClWRKY70 interacts with *ClACO5* in response to CGMMV infection. A yeast one-hybrid assay (Y1H) confirmed that ClWRKY70 could bind to the W-box element in the *ClACO5* promoter (**Figure 5B**). We also conducted a GUS transactivation experiment in *N. benthamiana* leaves to further validate this interaction. Leaves co-transformed with pCNF-ClWRKY70, and ClACO5pro-GUS exhibited significantly higher GUS activities than leaves infiltrated with the empty pCNF effector and ClACO5pro-GUS, as shown by the deeper blue color staining image and higher expression level of the *GUS* gene (**Figure 5D**). 

To further determine the interaction between ClWRKY70 and the *ClACO5* promoter, we carried out a dual-luciferase reporter assay. The binding of ClWRKY70 to the *ClACO5* promoter led to a nearly three-fold increase in the relative LUC/REN ratio vs. the control (**Figure 5C**). To verify the role of ClWRKY70 in regulating the expression of *ClACO5* in watermelon, we transiently transformed the leaves of 7-day-old seedlings with the pCNF-ClWRKY70 construct or empty vector (pCNF-EGFP). *ClWRKY70* transcript levels were significantly higher in leaves transformed with the pCNF-ClWRKY70 construct vs. the empty vector control, and the transcript levels of *ClACO5* increased nearly two-fold in leaves transformed with pCNF-ClWRKY70 vs. the pCNF-EGFP control (**Figure 5A**). Collectively, our results implied that ClWRKY70 functions as a transcriptional activator of *ClACO5* and promotes its transcription by binding to the *ClACO5* promoter.

### *ClWRKY70* enhances systemic acquired resistance to CGMMV infection

**Figure 6:**
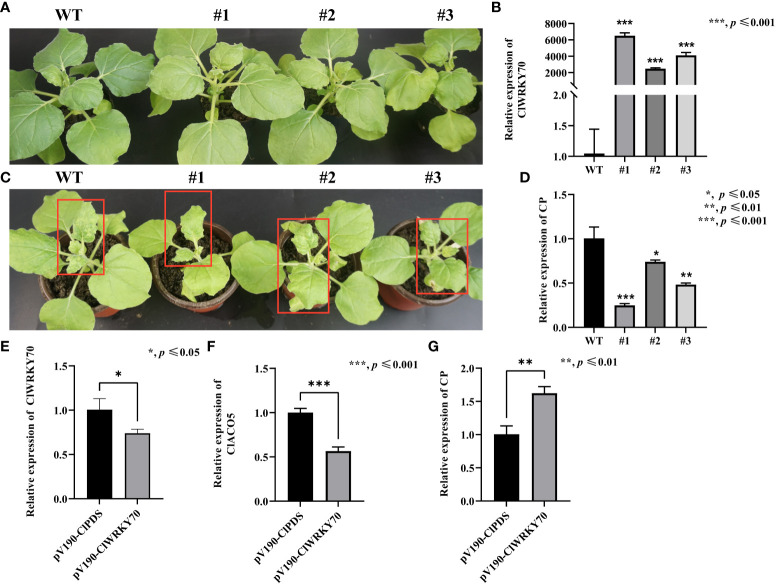
Knockdown and overexpression of ClWRKY70 enhance CGMMV susceptibility and resistance in watermelon plants and transgenic *N. benthamiana*. **(A)** Phenotype of WT and transgenic *N. benthamiana* plants under normal growth conditions. **(B)** The transcript levels of *ClWRKY70* in transgenic and WT *N. benthamiana* plants by RT-qPCR analysis. **(C)** Phenotype of transgenic and WT *N. benthamiana* plants under CGMMV infection conditions. **(D)** CGMMV CP accumulations in transgenic and WT *N. benthamiana* plants by RT-qPCR analysis. **(E-G)** The transcript levels of *ClWRKY70* **(E)**, *ClACO5* **(F)**, and CGMMV RNA accumulations **(G)** in pV190-ClPDS- and pV190-ClWRKY70-treated watermelon leaves at 20 dpi by RT-qPCR analysis. Watermelon and *N. benthamiana* plants were all collected at 20 dpi and 10 dpi, respectively. Values are means ± SD (n = 3). *p ≤ 0.05, **p ≤ 0.01, and ***p ≤ 0.001 (Student’s *t*-test).

Since ClWRKY70 participated in plant responses to *Acidovorax citrulli* infection (Zhang et al., 2020), and ClWRKY70 is an upstream regulator of *ClACO5*, we reasoned that ClWRKY70 might promote systemic acquired resistance to CGMMV infection. Next, we generated ClWRKY70-overexpressing transgenic *N. benthamiana* plants and selected three independent lines that had significantly higher *ClWRKY70* expression levels than the WT plants (**Figure 6B**). Under normal growth conditions, the WT and transgenic plants did not display phenotypic differences (**Figure 6A**). However, after CGMMV infection, transgenic plants exhibited milder mosaic and mottle symptoms in systemically infected leaves compared to WT plants (**Figure 6C**). In addition, WT plants had higher CGMMV CP transcript levels than transgenic plants (**Figure 6D**). The aforementioned results suggest that the heterologous expression of ClWRKY70 enhanced CGMMV resistance.

To further probe the function of ClWRKY70 in regulating the host’s susceptibility to CGMMV, we used the pV190 VIGS system to silence *ClWRKY70* in watermelon plants. Knockdown of *ClWRKY70* resulted in the simultaneous downregulation of *ClACO5* (**Figures 6E, F**). Finally, we analyzed CGMMV accumulation in *ClWRKY70-*knockdown and control (*PDS-*silenced) watermelon plants by RT-qPCR, finding that it was 62% higher in the *ClWRKY70*-knockdown plants (**Figure 6G**). This indicated that the knockdown of *ClWRKY70* increased the susceptibility of watermelons to CGMMV infection.

## Discussion

### ET production and *ClACO5* expression are induced in response to CGMMV infection

Plants have developed complex systems to defend themselves against viral attacks, in which phytohormones play essential roles in facilitating defense signaling in plants (Mishra et al., 2020). ET plays a dual role in plant-virus interactions; it promotes resistance in some interactions and contributes to disease progression in others (Chen et al., 2013; Zhu et al., 2014b; Zhao et al., 2017). For example, the rice dwarf virus (RDV) increases ET production by interacting with OsSAMS1, an essential component of the ET biosynthesis, resulting in an increase in the enzyme’s activity, thereby increasing susceptibility to RDV (Zhao et al., 2017). Southern rice black-streaked dwarf virus (SRBSDV) encoded P6 protein subcellular localization is changed during different stages of SRBSDV infection differentially altering ethylene signaling to support SRBSDV infection and transmission (Zhao et al., 2022). In the current study, we observed a marked increase in ET production in watermelon plants after CGMMV infection. When exogenous ET was applied prior to the mechanical inoculation of CGMMV, watermelon leaves had milder disease symptoms, lower disease indices, and reduced virus accumulation compared with mock plants (**Figure 1**). These results imply that ET accumulation in watermelon plants might promote resistance to CGMMV infection. The opposite roles of ET in plant responses to viral infection might be related to different virus-host combinations as well as crosstalk between ET and other phytohormones.

ACS and ACO are major enzymes that catalyze the two key steps of ET biosynthesis (Kim et al., 2003). Eight *ACS* and eight *ACO* genes of watermelon plants were previously identified (Zhou et al., 2016). In the current research, we found the expressions of three *ClACO* genes (Cla97C07G140100, Cla97C07G144530, and Cla97C10G205440) were altered by CGMMV infection by analyzing the transcript levels of these genes under CGMMV infection. Among them, only Cla97C07G144530 (*ClACO5*) was upregulated. Whereas the transcript levels of all *ClACS* genes were unchanged (**Supplementary Figure S1**). Thus, *ClACO* genes play more important roles than *ClACS* genes in the response of watermelon plants to CGMMV infection.

The expression pattern of *ClACO5* is largely consistent with the changes in ET levels in watermelon leaves during CGMMV infection (**Figure 3C**), and ClACO5 shares high sequence similarity with CuACO5 and AtDMR6 (**Supplementary Figure S2**). Based on these results, we speculate that ClACO5 may be vital for ET production during CGMMV infection in watermelon leaves. *AtDMR6* encodes a putative 2OG-Fe(II) oxygenase associated with defense responses but required for susceptibility to downy mildew (Zeilmaker et al., 2015); it also participates in plant responses to bacteria and oomycetes, flavonoid biosynthesis, and SA catabolism (Falcone Ferreyra et al., 2015; Zhang et al., 2017), but it is unclear whether this gene participates in plant responses to viruses. In the present study, the knockdown of *ClACO5* in watermelons conferred enhanced susceptibility to CGMMV infection, whereas heterologous overexpression of this gene in *N. benthamiana* enhanced resistance to CGMMV infection (**Figure 3**). Taken together, we conclude that *ClACO5* functions in plant responses to CGMMV infection and enhances CGMMV resistance.

### ClWRKY70 directly activates the expression of *ClACO5*

WRKY TFs regulate gene expression by binding directly to the W-box in their target genes’ promoters (Rushton et al., 2010). WRKY70s in some species, such as *Arabidopsis*, wheat (*Triticum aestivum*), and tomato (*Solanum lycopersicum*), play vital roles in plant responses to pathogen infection (Atamian et al., 2012; Wang et al., 2017; Zhou et al., 2018; Liu et al., 2021). However, far less is known about their roles in defense against viral pathogens as compared to fungal or bacterial pathogens. In the present study, we report that *ClWRKY70* is induced by CGMMV infection. We observed a marked difference between *ClWRKY70*-overexpressing transgenic *N. benthamiana* and WT plants in leaves systemically infected with CGMMV, suggesting that ClWRKY70 might inhibit CGMMV accumulation.

*WRKY70* regulates the expression of numerous defense-related genes (Machens et al., 2014), including *SARD1*, *PR1*, *PR2*, and *PR5 (* Li et al., 2004). WRKY70 may also inhibit ET- and JA-responsive gene expression (Li et al., 2004; Li et al., 2006; Li et al., 2019), but whether it regulates the expression of genes related to phytohormone biosynthesis remains unresolved. In the current study, we found that both *ClWRKY70* and *ClACO5* were consistently upregulated after CGMMV infection (**Figures 1C**, **4A**). *ClWRKY70* encodes a TF that positively regulates watermelon resistance to CGMMV (**Supplementary Figures 4**, **6**). We also showed that the *ClACO5* promoter contains a WRKY-binding site. ClWRKY70 directly bound to the *ClACO5* promoter and enhanced its activity in *N. benthamiana* leaves and upregulated this gene in watermelon leaves (**Supplementary Figure S4** and **Figure 5**), implying that *ClACO5* is a target gene of ClWRKY70. Based on these results, we propose that ClWRKY70 functions as a major positive regulator of ET biosynthesis by activating *ClACO5* expression in watermelon leaves and contributes to the resistance of watermelon plants to CGMMV infection.

SA can promote *ClWRKY70* expression (Zhang et al., 2020), and we previously determined that CGMMV infection can induce SA accumulation and that treatment with exogenous SA significantly enhances the CGMMV resistance of watermelons (Liu et al., 2023a). Based on these and the current findings, we propose that SA affects ET biosynthesis by inducing the expression of *ClWRKY70* and therefore activation of ClACO5 enhanced resistance of watermelon plants to CGMMV infection. In addition, we previously showed that SA-induced flavonoid biosynthesis plays a vital role in watermelon development and CGMMV resistance (Liu et al., 2023a), whereas AtDMR6, which is highly homologous to ClACO5, has flavone synthase activity (Falcone Ferreyra et al., 2015). Therefore, it would be worth investigating whether SA-induced flavonoid biosynthesis also involves the regulation of *ClACO5* expression by ClWRKY70 in watermelon leaves defense against CGMMV infection and whether SA, ET, and flavonoids form a complex transcriptional regulatory network during the defense response of watermelons against CGMMV. Furthermore, we previously found that ET could induce ClAGO5 expression, thus speculating that ethylene may enhance watermelon defense against CGMMV via activating RNA silencing pathway (Liu et al., 2023b).

**Figure 7:**
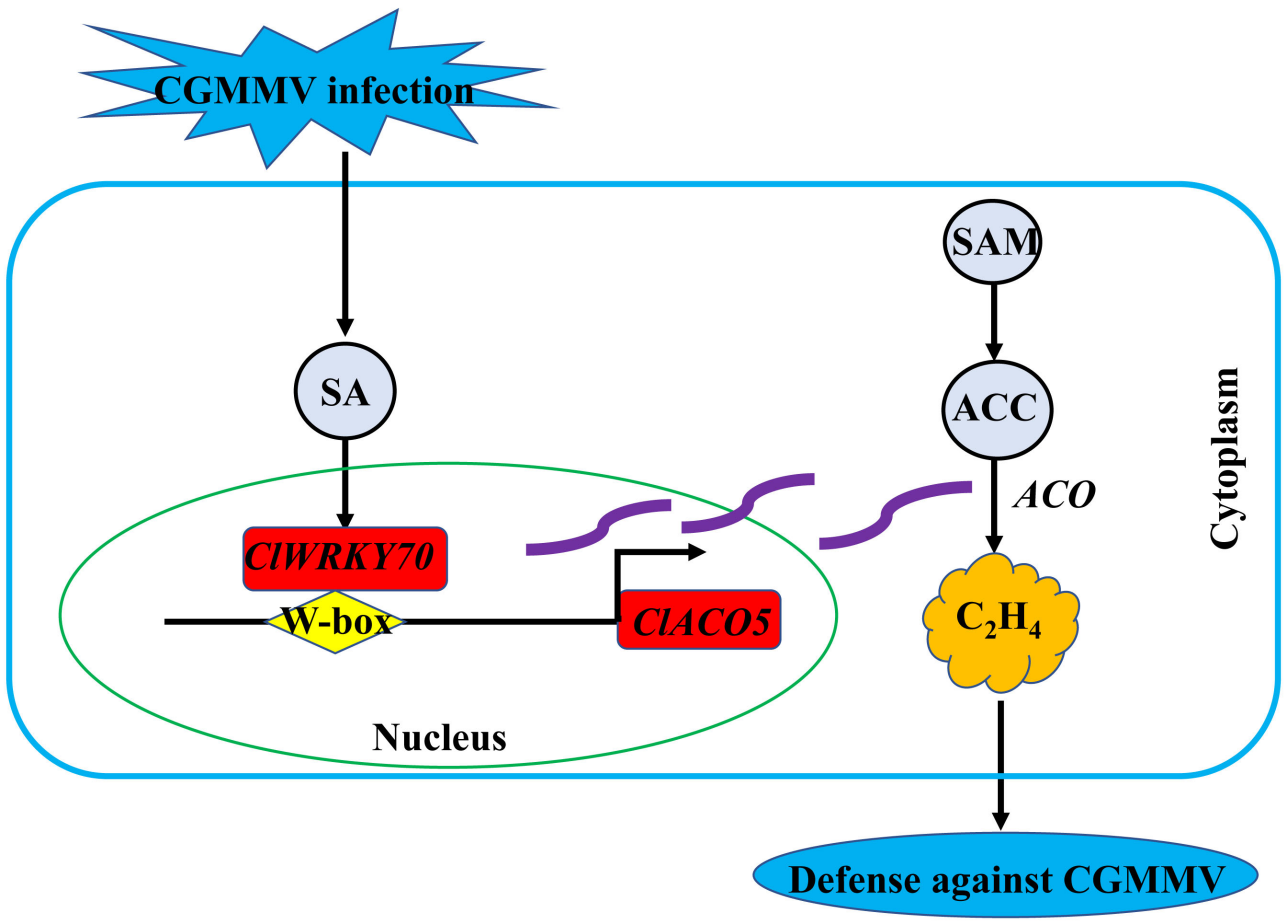
A proposed working model illustrating the regulatory role of SA responsive *ClWRKY70*-*ClACO5* module in ethylene accumulation under CGMMV infection conditions. CGMMV infection increases endogenous SA, which subsequently triggers the SA signaling transduction pathway. *ClWRKY70*, involved in SA signaling, positively regulates *ClACO5* by directly binding to the W-box element and activating the promoter, resulting in the upregulation of *ClACO5*, which is then integrated into the metabolic pathway for promoting ethylene biosynthesis. The *ClACO5* proteins are shown using the purple wavy lines.

Taken together, our results demonstrate that ClACO5, localized to the nucleus and cytoplasm, and involved in ET biosynthesis, enhances CGMMV resistance. We propose that CGMMV infection increases SA biosynthesis in watermelon plants, resulting in the activation of downstream signaling pathways. *ClWRKY70*, involved in SA signaling, regulates *ClACO5* expression by interacting with the W-box element in its promoter, thereby increasing ET levels (**Figure 7**). This model unveils the molecular mechanisms and transcriptional regulatory network of ET accumulation in response to CGMMV infection.

## Materials and methods

### Plant materials and treatments

The susceptible watermelon variety Zhengkang No. 2 and tobacco (*N. benthamiana*) were used in this research work. To analyze the effects of exogenous ET and ACC on plant responses to CGMMV, 7-day-old watermelon seedlings were sprayed with water, 500 μM ET, or 2 mM ACC for 3 days before CGMMV inoculation. Three biological replicates of leaves were collected 10 days after CGMMV treatment and used for RNA extraction.

### Measuring ET production

ET production was measured according to a previous method (Kawano and Shimokawa, 1994). Approximately 5 g of leaf tissue was placed into a glass bottle. The bottle was sealed and incubated at room temperature for at least 5 hours. A 1ml sample of gas was removed from the bottle and analyzed by gas chromatography (Shimadzu GC-2010).

### Detection of ACC

Phytohormones ACC contents were detected by MetWare (http://www.metware.cn/) based on the AB Sciex QTRAP 6500 LC-MS/MS platform.

### Sequence analysis of *ClACO5*

Multiple alignment of the putative protein sequences encoded by *ClACO5* and its homologs was performed using ClustalW software in conjunction with MEGA 7.0; then, the molecular weight and theoretical isoelectric point of the deduced ClACO5 protein was predicted using ExPASy (http://www.expasy.org), an online tool. The NewPLACE (https://www.dna.affrc.go.jp/PLACE/) database was used to identify the *cis*-elements in the *ClACO5* promoter.

### RNA extraction and RT-qPCR analysis

Total RNAs from watermelon leaf tissues were extracted using the method listed in the kit manual (Tiangen, Beijing, China). After the first-strand cDNA was synthesized, five-fold diluted cDNA products were subjected to qPCR using SYBR Green Master Mix (Vazyme, Nanjing, China) on a Roche Real-Time PCR system. We analyzed gene expression levels by the 2^–△△CT^ method (Livak and Schmittgen, 2001). The primers used for RT-qPCR are included in **Supplementary Table S2**.

### Subcellular localization of ClACO5 and ClWRKY70

The *ClACO5* and *ClWRKY70* coding sequences (CDSs) without the stop codons were amplified via 2×Fast Pfu Master Mix (novoprotein, Suzhou, China) and cloned into the pCNF-GFP vector. The recombinant 35S-ClACO5-GFP, 35S-ClWRKY70-GFP, and 35S-GFP (control) plasmids were transferred into the *A.tumefaciens* strain GV3101. *N. benthamiana* leaves were infiltrated with the bacterial suspensions, together with a plasmid expressing nuclear or membrane markers, 35S-H2B-mCherry or 35S-OsMCA1-mCherry (OD600~0.6). We observed the subcellular localizations of 35S-H2B-mCherry, 35S-OsMCA1-mCherry, 35S-ClACO5-GFP, 35S-ClWRKY70-GFP, and 35S-GFP under a confocal laser-scanning microscope.

### Transcriptional activation assay

The *ClWRKY70* CDS was cloned into the pGBKT7 vector. Yeast strain AH109 cells were transformed with the resulting construct and plated on SD/–Trp, SD/–His/–Trp/–Ade, or SD/–Trp/–Ade/–His supplemented with X-α-Gal. The pGBKT7 + pGADT7-T vector was used as a negative control.

### Yeast one-hybrid assay

The *ClWRKY70* CDS was ligated into the pGADT7 vector. The 789-bp promoter region containing a W-box *cis*-element was amplified with primers AD-ClWRKY70-F/R. Then, the amplified fragment was ligated into the pAbAi vector to generate the bait construct. An interaction assay of the *ClWRKY70* and *ClACO5* promotor fragments was conducted using a Matchmaker™ Gold Yeast One-Hybrid Library Screening System Kit (Clontech, San Francisco, USA).

### GUS and dual luciferase reporter assays

The PCR-amplified 789-bp fragment of the *ClACO5* promoter was cloned into pCAMBIA3301 and pGreenII 0800-LUC to generate reporter constructs. The *ClWRKY70* CDS was cloned into pCNF to produce the effector construct. The reporter and effector constructs were transferred into *A. tumefaciens* GV3101 with the pSoup helper vector and *A. tumefaciens* strain GV3101, respectively, which were then used to coinfect 4-week-old tobacco leaves. At 48-72 h after infiltration, LUC and REN activities and GUS activity were measured in the leaves of these plants.

### Transient expression in watermelon leaves

The expression construct pCNF-ClWRKY70 was transferred into *A. tumefaciens* GV3101; the empty vector pCNF was used as a negative control. Watermelon leaves were infiltrated with these cultures, collected 3 days after infiltration, and used to analyze the expression level of *ClWRKY70* and *ClACO5*.

### VIGS

A 300-bp fragment of *ClACO5* or *ClWRKY70* was amplified with gene-specific primers pV190-ClWRKY70-F/R or pV190-ClACO5-F/R (**Supplementary Table S2**), and cloned into pV190. Then, according to the previous description, all recombinant plasmid were separately transformed and injected into watermelon leaves (Liu et al., 2020). At 20 dpi, systemically infected leaves were collected for qPCR and RNA gel blot analyses.

### Stable heterologous expression of *ClACO5* or *ClWRKY70* in *N. benthamiana*

The *ClACO5* or *ClWRKY70* CDS was amplified and ligated into the expression vector pBWA(V)HS-ccdB-GFP (Biorun, Wuhan, China). The *A. tumefaciens* strain GV3101 harboring the *ClACO5* or *ClWRKY70* ORF recombinant plasmids was transformed into *N. benthamiana*. We selected three positive T_2_ transgenic lines for further experiments.

### Evaluation of the CGMMV resistance of *ClACO5*- or *ClWRKY70*-overexpressing

***N. benthamiana* plants**

*N. benthamiana* plants from the WT line and three overexpressed ClACO5 transgenic lines (OE#4, OE#9, and OE#21) or three overexpressed ClWRKY70 transgenic lines (OE#1, OE#2, and OE#3) were inoculated with CGMMV. At 10 dpi, the plants were observed, and leaves were collected for measurement of target gene expression and CGMMV accumulation.

### RNA gel blot analysis

The leaves of watermelon plants from the VIGS experiment were collected, and total RNA was extracted and used to analyze CGMMV accumulation with a DIG Northern Starter kit (Roche, Mannheim, Germany). The detailed method refers to a previously described method (Liu et al., 2017).

### Statistical analysis

The inoculations of CGMMV were repeated at least twice with at least three replicates for each line and each time point. GraphPad Prism 9.0 was used to process all data and analyze statistical differences using Student’s *t*-tests at significance levels of *p ≤* 0.05 (*), *p ≤* 0.01 (**), *p ≤* 0.001 (***), and *p ≤* 0.0001 (****).

## Data availability statement

The original contributions presented in the study are included in the article/**Supplementary Material**. Further inquiries can be directed to the corresponding authors.

## Author contributions

ML: Conceptualization, Data curation, Formal Analysis, Investigation, Methodology, Software, Validation, Writing – original draft. BK: Methodology, Writing – review & editing. HW: Methodology, Project administration, Writing – review & editing. BP: Project administration, Writing – review & editing. LL: Methodology, Writing – review & editing. NH: Resources, Supervision, Writing – review & editing. QG: Funding acquisition, Writing – review & editing.
